# Predictive value of fibrinogen-to-albumin ratio in early missed miscarriage: a case–control study

**DOI:** 10.3389/fmed.2026.1763764

**Published:** 2026-03-25

**Authors:** Wenhua Liu, Shanshan Tang, Miao Deng

**Affiliations:** Department of Obstetrics and Gynecology, Hangzhou Women’s Hospital, Hangzhou, China

**Keywords:** early missed miscarriage, fibrinogen to albumin ratio, neutrophil to lymphocyte ratio, prothrombotic state, inflammation

## Abstract

**Background:**

Currently, an increasing amount of evidence highlights the significant role that prothrombotic conditions play in the occurrence of early missed miscarriage.

**Objective:**

In this research project, our objective was to investigate the predictive function of the fibrinogen-to-albumin ratio (FAR) in patients with early missed miscarriage.

**Methods:**

In this study, a total of 180 women were enrolled, comprising 90 women who experienced early missed miscarriage and 90 women with normal pregnancies who chose to undergo elective abortion. A thorough comparison was made between the missed miscarriage group and the control group regarding demographic characteristics and several routine blood parameters, including fibrinogen, albumin, D-dimer, the fibrinogen-to-albumin ratio (FAR), the neutrophil-to-lymphocyte ratio (NLR), platelet count (PLT), the platelet-to-lymphocyte ratio (PLR), and mean platelet volume (MPV). Subsequently, receiver operating characteristic (ROC) curve analysis was performed to assess and quantify the predictive capacity of these parameters.

**Results:**

No significant discrepancies were observed in albumin, D-dimer, mean platelet volume (MPV), platelet count (PLT), and platelet-to-lymphocyte ratio (PLR) between the two cohorts (*p* > 0.05). Conversely, the neutrophil-to-lymphocyte ratio (NLR), fibrinogen, and the fibrinogen-to-albumin ratio (FAR) were elevated in the early missed miscarriage group compared with the control group (*p* < 0.05). Receiver operating characteristic (ROC) curve analysis revealed that the NLR exhibited a sensitivity of 71.1% and a specificity of 74.4% with a cutoff value of 3.58, whereas the FAR exhibited a sensitivity of 90% and a specificity of 81.1% with a cutoff value of 0.008 for predicting missed miscarriage.

**Conclusion:**

Our research findings indicated that the FAR and NLR are effective parameters for predicting missed miscarriage, as demonstrated by their respective sensitivity and specificity.

## Introduction

Pregnancy loss is a common complication during pregnancy, and missed miscarriage is one of its clinical presentations. It is pathologically characterized by the death of the embryo or fetus that remains within the uterus during the first 20 weeks of gestation. Complications associated with missed miscarriage, such as uterine adhesions, secondary infertility, bleeding, and infections, pose a significant threat to women’s physical and mental health (1). The incidence of missed miscarriage in clinically diagnosed pregnancies is currently estimated to be approximately 8–15% (2). Multiple factors have been identified as potential risk factors for missed miscarriage, including advanced maternal age, adverse obstetric history, genetic factors, immune abnormalities, endocrine disorders, uterine anomalies, thrombophilia, and environmental influences. However, the precise pathophysiological mechanisms underlying this condition remain unclear (3).

Thrombotic conditions can disrupt the maternal–fetal circulation and impede placental development. They may lead to venous or arterial thrombosis, which could contribute to pregnancy loss (4). To identify individuals at risk, thrombophilia panels incorporating genetic analyses have been evaluated. Recently, there has been an increasing focus on identifying new and effective biomarkers to monitor individuals with a prothrombotic tendency (5, 6). In particular, routine blood parameters have been investigated in the context of vascular thrombosis (6). Moreover, many studies have explored the roles of thrombotic and inflammatory processes in miscarriage (7, 8). However, previous research has not extensively examined changes in standard laboratory markers, which are studied in other thrombotic and/or inflammatory conditions, in patients with missed miscarriage.

Our current study aimed to identify disparities in routine blood parameters, including fibrinogen, D-dimer, the fibrinogen-to-albumin ratio (FAR), the neutrophil-to-lymphocyte ratio (NLR), platelet count, and mean platelet volume (MPV), between the missed miscarriage group and the control group.

## Materials and methods

### Data source and collection

This study was approved by the ethics committee of the hospital. The study was conducted according to the principles of the Declaration of Helsinki and the International Conference on Harmonization Guidelines for Good Clinical Practice. The sample size was determined by the availability of eligible patients during the study period. A *post-hoc* power analysis using G*Power software confirmed that the sample size was adequate to detect significant differences in the primary predictive marker (FAR). From December 2018 to December 2020, 90 women with normal pregnancies terminated by artificial abortion and 90 women with early missed miscarriage were enrolled in the Department of Gynecology Clinic at Hangzhou Women’s Hospital. All enrolled participants were aged between 18 and 35 years, with a gestational age ≤12 weeks and singleton pregnancies.

The inclusion criteria of the study group were patients with missed miscarriage diagnosed by ultrasound, including cases in which the length of the head and the hip was ≥7 mm with no fetal heartbeat detected, the average diameter of the pregnancy sac in the uterine cavity was ≥25 mm with no visible embryo, there was absence of a yolk sac in an intrauterine pregnancy or no embryo and fetal heartbeat detected after 2 weeks, or the yolk sac was present in intrauterine pregnancy but no fetal heartbeat was detected after 11 days. The inclusion criteria of the control group were women with normal pregnancies who underwent artificial abortion. Exclusion criteria included patients with incomplete data, chromosomal abnormalities, uterine structural abnormalities, a history of recurrent miscarriage, acute or chronic infectious diseases, or cancer; patients receiving progesterone therapy or any other medication for underlying medical conditions; and women who smoked during pregnancy.

In the first trimester, demographic and laboratory parameters were collected from patient records. Basic information, including age, gestational week, gravida, parity, body weight, and height, was recorded. Routine blood counting parameters, including neutrophil [%] and lymphocyte [%] to calculate the NLR [%], albumin [g/dL] and fibrinogen [μg/mL] to calculate the FAR [%], D-dimer [μg/L], MPV [fl], and platelet count [10–3/μL], were recorded. Preoperative parameters, such as complete blood count (CBC), were compared between the missed miscarriage and control groups.

### Statistical analysis

Statistical analyses were performed using SPSS version 23.0. The Kolmogorov–Smirnov test was applied to assess the normality of continuous variables. For variables with a normal distribution, data were presented as mean ± standard deviation (SD), and comparisons between groups were conducted using the independent samples *t*-test. For non-normally distributed variables, data were expressed as median with interquartile range (IQR), and the Mann–Whitney *U*-test was used. Homogeneity of variances was assessed using Levene’s test; if the assumption of equal variances was violated, Welch’s correction was applied. Categorical variables were presented as counts and percentages, and comparisons were performed using the chi-squared test or Fisher’s exact test, as appropriate. Optimal cutoff values for the significant parameters were determined using the receiver operator characteristic (ROC) curve analysis. The area under the curve (AUC) was used to evaluate the sensitivity, specificity, and accuracy of each test. Variables were considered statistically significant at a *p*-value of < 0.05.

## Results

A total of 180 patients with missed miscarriage were compared with 180 participants in the control group with normal pregnancies. The two groups showed no significant differences in maternal age, gestational age, body mass index (BMI), gravidity, or parity (*p* > 0.05), as detailed in Table 1.

**Table 1:** Demographic and clinical features of the groups.

Variables	Missed miscarriage (*n* = 90)	Controls (*n* = 90)	*p*
Age (years)**	28.56 ± 2.75	28.03 ± 3.97	0.307
BMI (kg/m^2^) **	23.11 ± 4.3	23.05 ± 4.1	0.88
Gestational weeks*	8 (7–9)	8 (7–9)	0.675
Gravida*	2 (1–3)	2 (1–3)	0.103
Parity*	1 (0–1)	1 (0–1)	0.096

Data are presented as mean ± standard deviation for normally distributed variables**, or median (interquartile range, Q1–Q3) for non-normally distributed variables*.

When comparing blood parameters between the groups, fibrinogen, the FAR, and the NLR differed significantly between the missed miscarriage and control groups (*p* < 0.001). In contrast, albumin, D-dimer, MPV, PLT, and the PLR were similar in both groups (*p* > 0.05). A summary of the comparison between demographic characteristics and blood sample variables is provided in Table 2.

**Table 2:** Comparison of laboratory values between two groups.

Variables	Missed miscarriage (*n* = 90)	Controls (*n* = 90)	*p*
Fibrinogen, g/L	4.28 ± 0.68	3.05 ± 0.58	<0.0001
Albumin, g/dL	42.50 ± 2.43	42.48 ± 2.27	0.957
FAR	0.10 ± 0.01	0.07 ± 0.01	<0.0001
NLR	3.63 ± 2.03	2.91 ± 1.01	0.003
PLR	132.09 ± 36.52	148.47 ± 95.58	0.184
MPV, fl	9.41 ± 1.28	9.51 ± 1.14	0.846
PLT	231.37 ± 53.06	243.08 ± 48.36	0.266
D-dimer, μg/L	500.22 ± 679.65	493.88 ± 387.44	0.939

FAR, fibrinogen-to-albumin ratio; NLR, neutrophil to lymphocyte ratio; PLR, platelet to lymphocyte ratio; MPV, main platelet volume.

**Figure 1:**
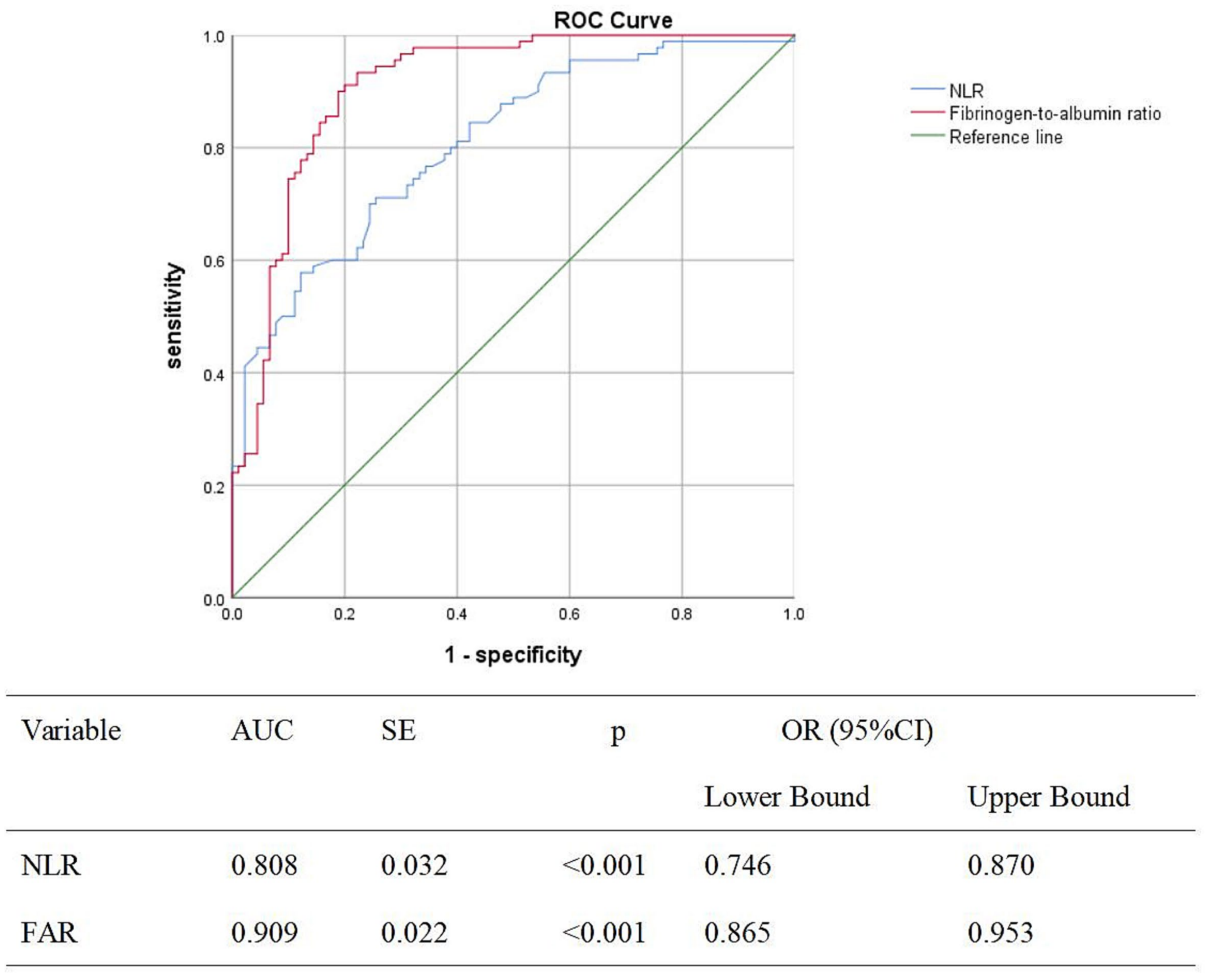
ROC curves, AUC values, standard error, *p*-value, and 95% CI of the AUC of the NLR and the FAR.

In the ROC curve analysis (Figure 1), FAR values above the reference line were associated with increased risk. A cutoff value of 0.008 predicted the risk of abortion with a sensitivity of 90% and a specificity of 81.1% (AUC: 0.909, 95% CI: 0.865–0.953). Additionally, NLR exhibited a sensitivity of 71.1% and a specificity of 74.4% at a cutoff value of 3.58. The ROC curve results for the NLR and FAR are presented in Figure 1.

## Discussion

In the current investigation, no significant differences were observed in albumin, platelet count (PLT), mean platelet volume (MPV), D-dimer, or the platelet-to-lymphocyte ratio (PLR) between the missed miscarriage and control groups (*p* < 0.05). In contrast, fibrinogen, the fibrinogen-to-albumin ratio (FAR), and the neutrophil-to-lymphocyte ratio (NLR) were significantly elevated in the missed miscarriage group (*p* < 0.05). Further analysis based on the area under the curve (AUC) indicated that the FAR emerged as the most efficacious predictor of missed miscarriage, with an optimal cutoff value of 0.008, an AUC of 0.909, a specificity of 90%, and a sensitivity of 81.1%.

During early pregnancy, inflammation plays a crucial role in facilitating embryo implantation. However, an imbalance in the inflammatory state may lead to miscarriage (9). Initially, a mildly inflammatory environment is essential for successful embryo implantation. Subsequently, the local decidua must establish an anti-inflammatory and immune-tolerant microenvironment to ensure the embryo’s survival and development (10).

In recent years, given their convenience, simplicity, sensitivity, versatility, and rapidity, complete blood count (CBC) parameters, as systemic markers of inflammation, have increasingly captured attention in miscarriage research. Among these, the NLR and the PLR are frequently used as inflammation markers. Nevertheless, the relationship between the NLR, PLR, and miscarriage remains somewhat unclear. For instance, Biyik et al. (11) reported that both NLR and PLR values were higher in the missed miscarriage group than in healthy pregnant women. In contrast, Kim et al. (12) suggested that the NLR may serve as a prognostic determinant for differentiating between missed abortion and threatened abortion. Furthermore, the interpretation of the NLR during pregnancy is multifaceted. While an elevated NLR in our missed miscarriage cohort may reflect a systemic inflammatory imbalance contributing to pregnancy loss, it is important to acknowledge that the NLR can also physiologically increase during normal gestation. In addition to its role as an inflammatory marker, elevated NLR levels may indicate broader maternal vascular changes. For example, previous studies have shown that higher NLR levels in otherwise healthy pregnant women are independently associated with increased carotid intima-media thickness, indicating early subclinical arterial remodeling (13). Therefore, the elevated NLR levels observed in our study may represent a dual phenomenon: a contributor to, or a consequence of, prothrombotic and inflammatory states, and a marker of underlying maternal vascular vulnerability that could predispose to inadequate placental perfusion. This dual interpretation highlights the need for further research to delineate the specific pathophysiological pathways linking the NLR to missed miscarriage. However, in our study, no significant difference in the PLR was observed between the missed miscarriage and normal pregnancy groups (*p* > 0.05). This discrepancy may be attributable to methodological differences or variations in the equipment used to assess missed miscarriage cases. Further research is warranted to ascertain whether inflammation at the fetal-maternal interface reflects systemic inflammatory changes. Overall, our study suggests that the PLR does not have a decisive influence on the occurrence of missed miscarriage.

Fibrinogen (FIB) is intertwined with multiple factors. It demonstrates a pronounced correlation with platelet activation and fibrin formation and is also implicated in augmenting plasma viscosity (14). Additionally, FIB partakes in systemic inflammatory responses and instigates the secretion of cytokines and chemotactic factors (15). In contrast, albumin (ALB), functioning as a negative inflammatory biomarker, encompasses diverse capabilities such as anti-apoptotic, antioxidant, and anti-inflammatory properties (16). In light of prior reports documenting decidual fibrin deposition and enhanced fibrinolytic activity in the placental histopathology of miscarriage cases, we elected to examine the levels of fibrinogen and D-dimer in missed miscarriage patients. Our results revealed a conspicuously elevated fibrinogen level in these patients. However, the D-dimer level was comparable to that of the control group. The FAR, a novel inflammatory biomarker, has been adopted as a predictive indicator for venous and arterial disorders (15, 17, 18). Previous investigations have contended that fibrinogen and albumin can modulate blood viscosity and oncotic pressure, thereby contributing to the development of vascular thrombosis and inadequate perfusion (17, 18). Similarly, our study detected higher FAR levels in missed miscarriage patients compared with the control group. Cimsir et al. (19) reported elevated FAR levels in patients with recurrent pregnancy loss compared with the controls, and Usta et al. (20) demonstrated increased FAR levels in pregnancies with spontaneous abortions compared with healthy pregnancies, consistent with our findings. Furthermore, emerging research continues to identify novel protein biomarkers that may enhance our understanding of miscarriage pathophysiology. A recent study by Firatligil et al. (21) investigated the role of cysteine-rich angiogenic inducer 61 (CYR61), a matricellular protein involved in angiogenesis, cell adhesion, and tissue remodeling, in women with recurrent pregnancy loss. The authors reported significantly altered CYR61 levels in affected pregnancies, suggesting its potential involvement in impaired decidualization and placentation. While our study focused on systemic inflammatory and prothrombotic markers (NLR and FAR), the findings of Firatligil et al. (21) highlight the importance of the local endometrial microenvironment and vascular remodeling. Integrating such markers—reflecting both systemic inflammation (FAR and NLR) and local angiogenic dysfunction (CYR61)—could enable a more comprehensive multi-biomarker panel for risk stratification in early pregnancy. Future studies should explore the interplay between these pathways to better delineate the heterogeneous etiology of missed miscarriage.

Nonetheless, our study has several limitations. First, the sample size was relatively modest. Second, the study employed a retrospective design. Third, all cases were recruited solely from a single center. Additionally, certain biomarkers, such as cytokines and C-reactive protein, were not measured in serum samples, and chromosomal analysis was not performed on the aborted material.

## Conclusion

Our findings suggest that NLR and FAR levels may serve as potential predictors of missed miscarriage risk, given their high sensitivity and specificity. Notably, higher FAR levels may be associated with increased thrombotic activity in recurrent miscarriage. Larger cohort studies and comprehensive research are warranted to further investigate these associations and clarify the underlying mechanisms of serum fibrinogen, albumin, and elevated FAR levels.

## Data Availability

The original contributions presented in the study are included in the article/supplementary material, further inquiries can be directed to the corresponding author.
